# Supplementary figures and images for: Making a science out of preanalytics: An analytical method to determine optimal tissue fixation in real-time
Source: PLoS One. 2021 Oct 14;16(10):e0258495. doi: 10.1371/journal.pone.0258495 (PMC8516200; doi:10.1371/journal.pone.0258495)

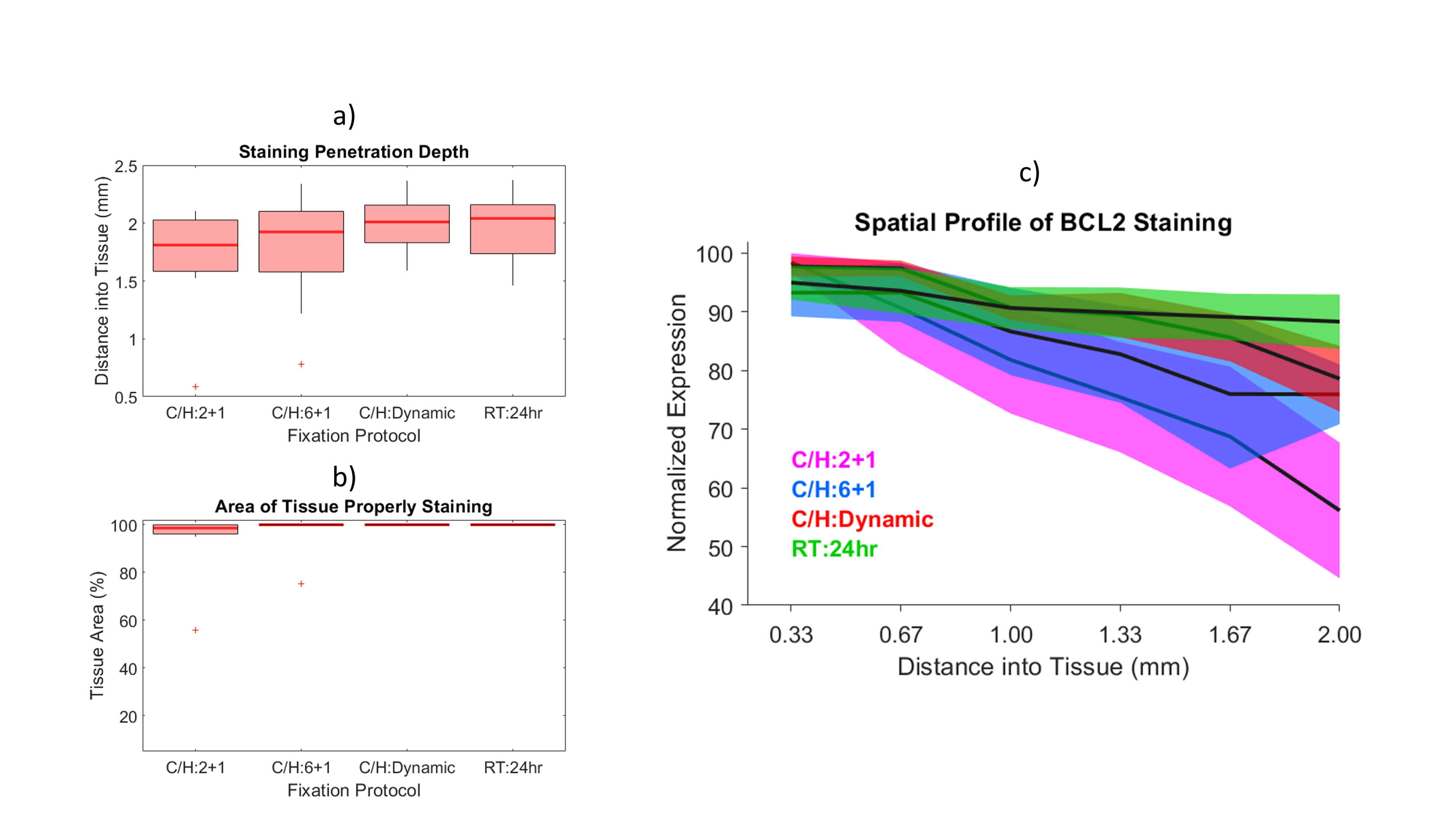

Supplement: S1 Fig — a) Penetration depth of proper bcl-2 staining. b) Area of proper bcl-2 staining. c) Normalized bcl-2 expression plotted versus distance into the tissue. (TIF) [file pone.0258495.s001.tif]

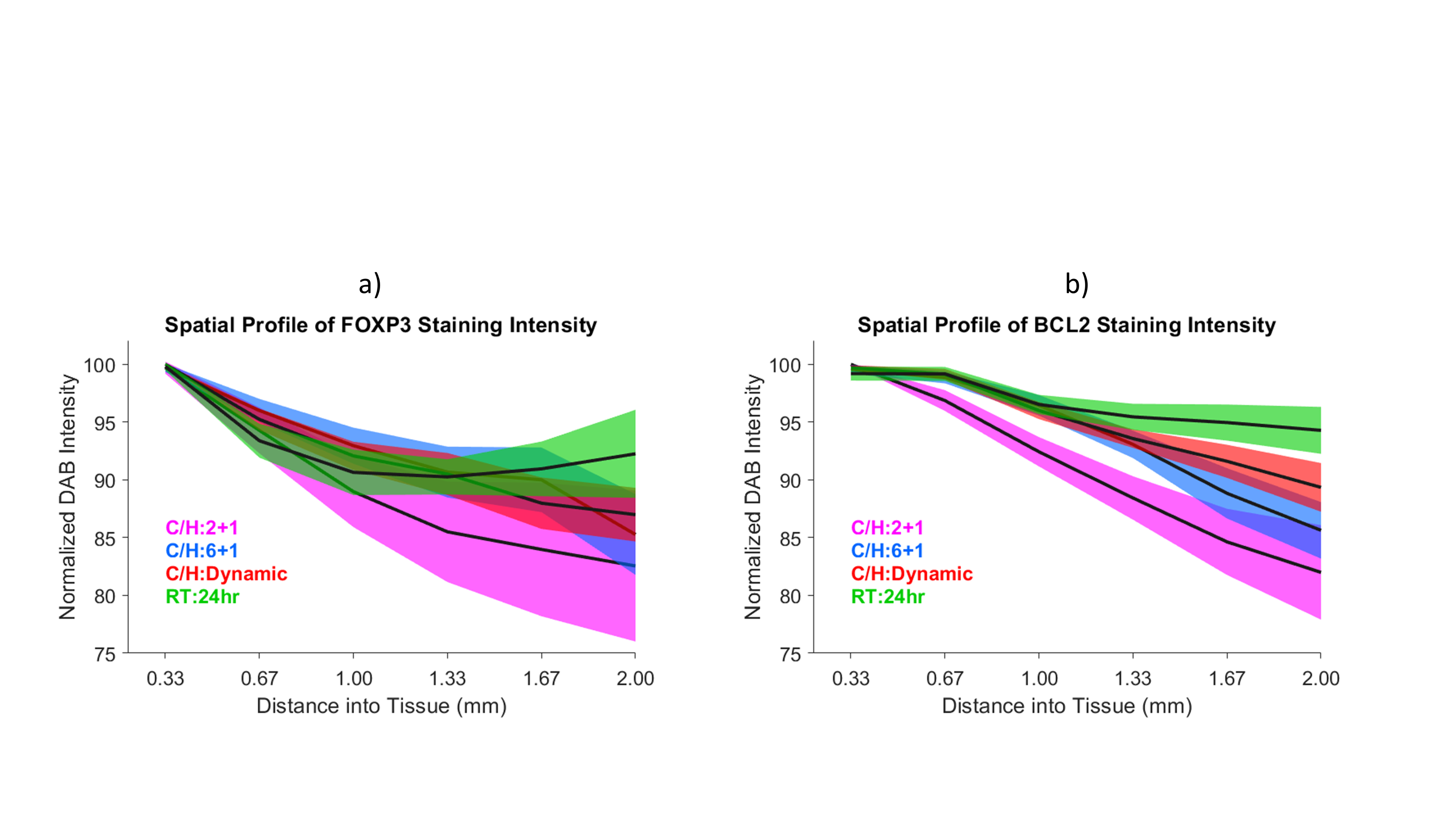

Supplement: S2 Fig — a) Normalized intensity of FOXP3 staining plotted versus distance into the tissue. b) Normalized intensity of bcl-2 staining plotted versus distance into the tissue. (TIF) [file pone.0258495.s002.tif]

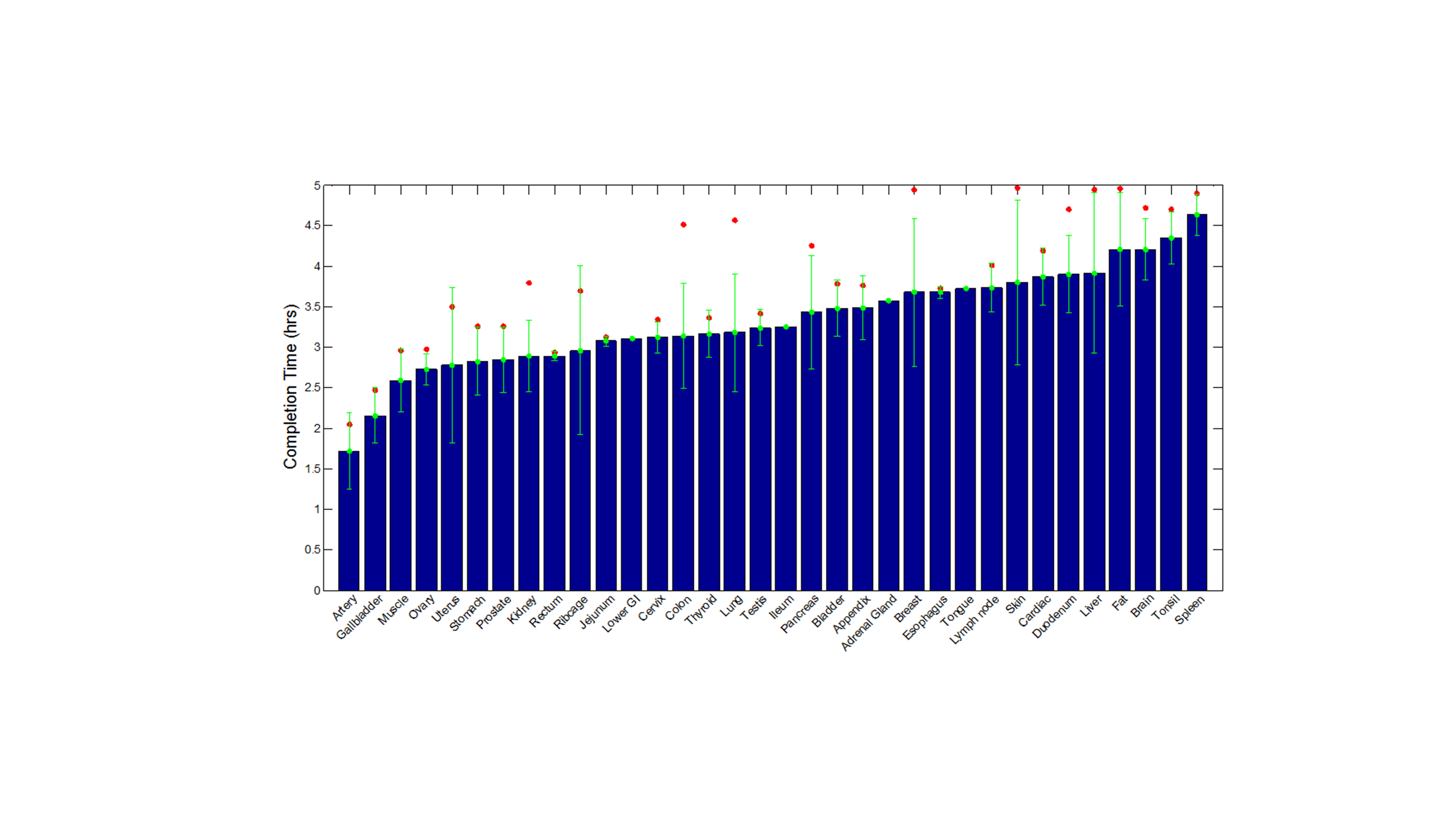

Supplement: S3 Fig — (TIF) [file pone.0258495.s003.tif]
