# Supplementary material for: Making a science out of preanalytics: An analytical method to determine optimal tissue fixation in real-time
Source: PLoS One. 2021 Oct 14;16(10):e0258495. doi: 10.1371/journal.pone.0258495 (PMC8516200; doi:10.1371/journal.pone.0258495)
Supplement: Attachment — Submitted filename: PONE-D-21-16557.docx [file pone.0258495.s006.docx]

The manuscript by Bauer and colleagues represents an interesting method for real-time evaluation of the tissue fixation process, using a digital acoustic interferometry algorithm that calculated the TOF differential followed by IHC digital pathology analysis on two markers. The work was performed on a single type of organ (tonsil) for a total of 87 samples in 4 different fixation methods. The analysis is based on different fixation times and temperatures, with deliberate hypofixation in some cases.

The real-time monitoring process allows to identify the moment in which the formalin penetration will allow to obtain the best morphological preservation result of the sample.

Both the technical and mathematical method are extremely accurate, as is the written and visual way of disseminating the results.

The English form is excellent, the statistics that are conducted in a rigorous manner.

The main problem is the structure of the experimental design, which consists of some important bugs.

1) Cases were collected from a single type of tissue.

Since different organs have different characteristics and fixation times, it would be appropriate to characterize other tissues. Similarly, the type of sampling (biopsy, surgical specimen, cytology) also has different fixation characteristics. Authors should consider completing the study with a pilot project on other tumor types and sampling

2) The analyzed markers are not classical membrane proteins. In order to understand if all cell compartments can benefit from this real-time fission assessment approach, it is recommended to try other markers.

3) Molecular analysis, and therefore the use of nucleic acids in pathological units, has become routine. An evaluation involving tissue fixation for FFPE samples cannot also evaluate the quality of the DNA. It is advisable to extract and qualify at least part of the cases evaluated using, for example, the bioanalyzer

4) The style of both the manuscript and the bibliography is completely wrong, and it does not follow the style of the journal. Please change and correct.
